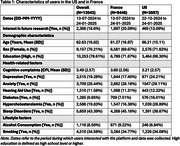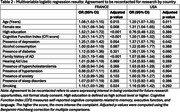# Docmemo: Transforming recruitment for clinical trials through an innovative screening platform in the US and France

**DOI:** 10.1002/alz70859_106552

**Published:** 2025-12-26

**Authors:** Federica Cacciamani, Graziella Mangin Laignel, Audrey Gabelle, Nicolas Beaume, Mahinthan Kengatharan, Igor Koval, Stanley Durrleman

**Affiliations:** ^1^ ARAMISLab, Sorbonne Université, Institut du Cerveau‐Paris Brain Institute‐ICM, CNRS, Inria, Inserm, AP‐HP, Hôpital de la Pitié Salpêtrière, Paris, France France; ^2^ Qairnel, Paris, France France; ^3^ Université de Montpellier, Montpellier France; ^4^ Memory Resource and Research Center of Montpellier, CHU de Montpellier, Hôpital Gui de Chauliac, Montpellier France

## Abstract

**Background:**

Recruiting participants for research studies on early‐stage Alzheimer’s disease (AD) is challenging yet crucial for advancing research and timely interventions. DocMemo, a digital platform for individuals with memory concerns in France (docmemo.fr) and the US (docmemo.com), provides cognitive screening, helps schedule memory‐related visits, and allows users to express interest in research participation.

We evaluated how effectively DocMemo can help identify potential research participants by examining how many users agreed to be recontacted, and factors associated with this agreement overall and within each country.

**Method:**

Demographic, cognitive, psychological, health‐related, and lifestyle data, as well as country, were collected from users interacting with DocMemo (i.e., completing cognitive health questionnaires or scheduling medical visits). All users were asked whether they agreed to be recontacted for future research opportunities (yes/no). Multivariable logistic regressions were used to identify factors linked to this agreement within each country, accounting for multiple comparisons.

**Result:**

We recorded 13,043 unique users (France: 9,446 from 13‐07‐2024 to 24‐01‐2025; US: 35,97 from 22‐10‐2024 to 24‐01‐2025). Their characteristics are detailed in Table 1.

The proportion of users agreeing to be recontacted for future research was 20.09% in France and 13.09% in the US.

In both countries, agreement was significantly associated with older age (France: OR=1.08, p=0.015; US: OR=1.20, p=0.011), more cognitive complaints (CFI score, France: OR=1.20, p<0.001; US: OR=1.20, p=0.002), and depression (France: OR=1.25, p=0.004; US: OR=1.37, p=0.042). In France, agreement was also higher among females (OR=1.19, p=0.008), those with high education (OR=1.52, p<0.001), and those with a family history of AD (OR=1.33, p<0.001). Other factors were not significantly associated with agreement in either country (p>0.05, Table 2).

**Conclusion:**

DocMemo provides a steady stream of individuals at risk for ADRD who express interest in research participation. This pool of potential participants can help meet recruitment needs for multiple studies simultaneously.